# Effects of Growth Temperature on the Compositions of GaAsSb/AlAsSb Epitaxial Layers Grown on GaAs Substrates

**DOI:** 10.1021/acsomega.6c03647

**Published:** 2026-08-18

**Authors:** Tsimafei Laryn, Yeonhwa Kim, Rafael Jumar Chu, Won Jun Choi, Daehwan Jung

**Affiliations:** † Center for Quantum Technology, 58975 Korea Institute of Science and Technology, Seoul 02792, South Korea; ‡ Nanoscience and Technology, KIST School, University of Science and Technology, Seoul 02792, Republic of Korea; § Department of Materials Science and Engineering, Korea University, Seoul 02841, South Korea

## Abstract

Sb-based III–V semiconductor materials are crucial for various applications in the short-wave and midwave infrared spectral ranges. However, epitaxial growth involving multiple group-V elements remains challenging because of their strong dependence on growth temperatures. In this work, we investigate the epitaxial growth of GaAsSb/AlAsSb layers on GaAs substrates under varying Sb fluxes and growth temperatures. We first identify an optimal growth window for AlAsSb to be between 580 and 620 °C, where smooth morphology and well-defined X-ray diffraction peaks are obtained. A systematic growth temperature study from 535 to 635 °C shows that the maximum Sb incorporation occurs at 585 °C. We additionally examine the different Sb incorporation rates and find that Al-containing layers incorporate significantly less Sb than their Ga-containing counterparts. This systematic study clarifies the temperature- and flux-dependent incorporation mechanisms in the GaAsSb/AlAsSb system and provides important details for the growth of high-quality GaAsSb and AlAsSb layers on GaAs for infrared photonic and optoelectronic applications.

## Graphical abstract

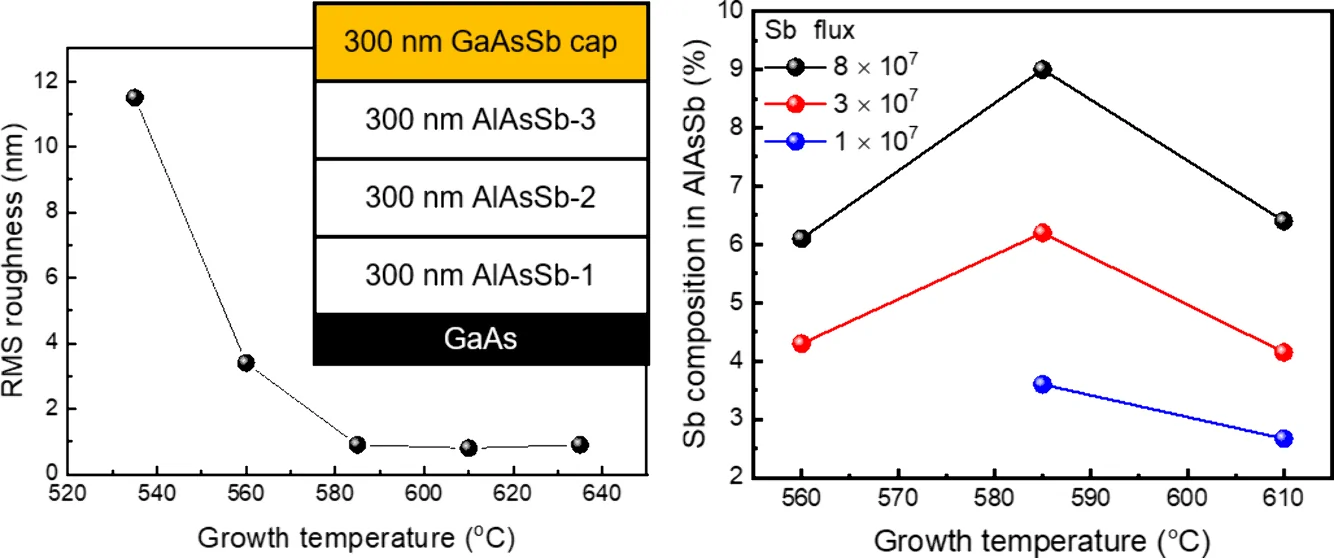

## 1 Introduction

Photodetectors operating in the short-wave and mid-wave infrared (SWIR and MWIR) spectral ranges are critical for a variety of civilian and military applications, including environmental monitoring, medical diagnostics and remote sensing.^1,2^ For decades, this field has been dominated by HgCdTe alloys because of their bandgap tunability and high device performance.^3^ However, HgCdTe material exhibits poor uniformity across large-area wafers and presents challenges for large-scale manufacturing.^4^

III–V semiconductors, particularly those incorporating As and Sb, have emerged as leading alternatives. These compound semiconductors offer a combination of low bandgap energies, high optical absorption coefficients, and high electron mobilities,^5,6^ while benefiting from the mature fabrication technologies developed for GaAs- and InP-based devices. Furthermore, their energy band structures can be engineered through careful control of alloy compositions, enabling a wide spectral tunability across the infrared wavelength range.^7,8^ Among the most prominent candidates are InAsSb, GaAsSb and AlAsSb, which have been successfully integrated into photonic and electronic devices.^9,10^

Among these materials, GaAsSb and AlAsSb have attracted particular attention.^11–15^ Their wide bandgap tunability and large band offsets make them especially suitable for applications such as electron-blocking layers in heterojunction devices and barrier layers in avalanche photodiodes.^16–18^ However, compared to InAsSb or GaAsSb, which have been the subject of extensive growth studies,^19,20^ the epitaxial growth of AlAsSb has been relatively unexplored. No comprehensive study of the optimal growth conditions has been reported, and the reproducibility of AlAsSb growth remains a key obstacle to its broader adoption in device structures.^21,22^ The main challenge arises from the difficulties in controlling the group-V composition. The incorporation coefficient of As is much higher than that of Sb. This is more crucial when the As fraction in an AlAsSb layer needs to be very low.^23^ This imbalance makes precise composition control difficult and hinders the growth of thick, high-quality AlAsSb layers. Furthermore, the group-V species incorporation ratio changes over different growth temperatures. These growth-related issues limit reproducibility and complicate the design of device structures that rely on sharp heterointerfaces and predictable band alignments. Lastly, investigating group-V incorporation dependency caused by different group-III elements, such as Ga or Al, is important for precise composition control of GaAsSb/AlAsSb epitaxial layers.

Compared to GaSb and InP, GaAs substrates offer lower cost, larger wafer sizes, and a more mature manufacturing technology. These advantages make GaAs an attractive platform for large-scale production of SWIR and MWIR optoelectronic devices. In addition, GaAs-based technologies are widely used in both research and industry, making the integration of GaAsSb and AlAsSb on GaAs particularly promising for future infrared optoelectronic devices. The different Sb incorporation behaviors between GaAsSb and AlAsSb layers for given As_2_ and Sb_2_ flux are also very important and systematically studied.

In this work, we address these challenges by systematically investigating the molecular beam epitaxy (MBE) of GaAsSb/AlAsSb layers on GaAs substrates. We first explore the optimum growth temperature of AlAsSb layers. The maximum Sb incorporation and high structural quality are achieved at 585 °C, as shown by reciprocal space mapping (RSM) and atomic force microscopy (AFM). We also define the growth window for AlAsSb layers to be 580–620 °C, where the high-quality films can be grown epitaxially. Furthermore, we find a significant 2-fold reduction in Sb incorporation efficiency when transitioning from Ga-containing to Al-containing ternary layers. By examining GaAsSb and AlAsSb growth over a wide temperature range from 535 to 635 °C, we provide practical information that can support the use of AlAsSb in infrared detectors and laser structures.

## 2 Experimental Details

The samples were grown in a Veeco Gen-930 MBE reactor on (001) semi-insulating GaAs substrates. The sample structure is shown in Figure 1(a). The growth rates used for all layers were fixed at 1.8 Å/s. The As V/III ratio was fixed at 18, while the Sb flux was varied from 1 × 10^–7^ Torr to 8 × 10^–7^ Torr, as shown in Figure 1(a). As_2_ and Sb_2_ were used as the group-V sources, with cracker temperatures of 900 and 800 °C, respectively. We first grew a GaAs buffer to restore the growth surface after oxide desorption. A 50 nm AlAs layer was grown on top to smooth the transition between GaAs and Al-containing ternary layers. The growth was followed by three 300 nm AlAsSb layers with different Sb flux values. Three AlAsSb layers grown with different Sb fluxes were intentionally incorporated into the structure to investigate the dependence of Sb incorporation on both growth temperature and As/Sb flux ratio. The use of multiple compositions within a single sample enables direct comparison of incorporation efficiencies under identical growth conditions. The As flux was fixed to be 3 × 10^–6^ Torr for all the Sb-containing layers, while the Sb flux varied. This was done to study the incorporation efficiency of Sb by controlling the group-V ratio only through the Sb flux.

**Figure 1:**
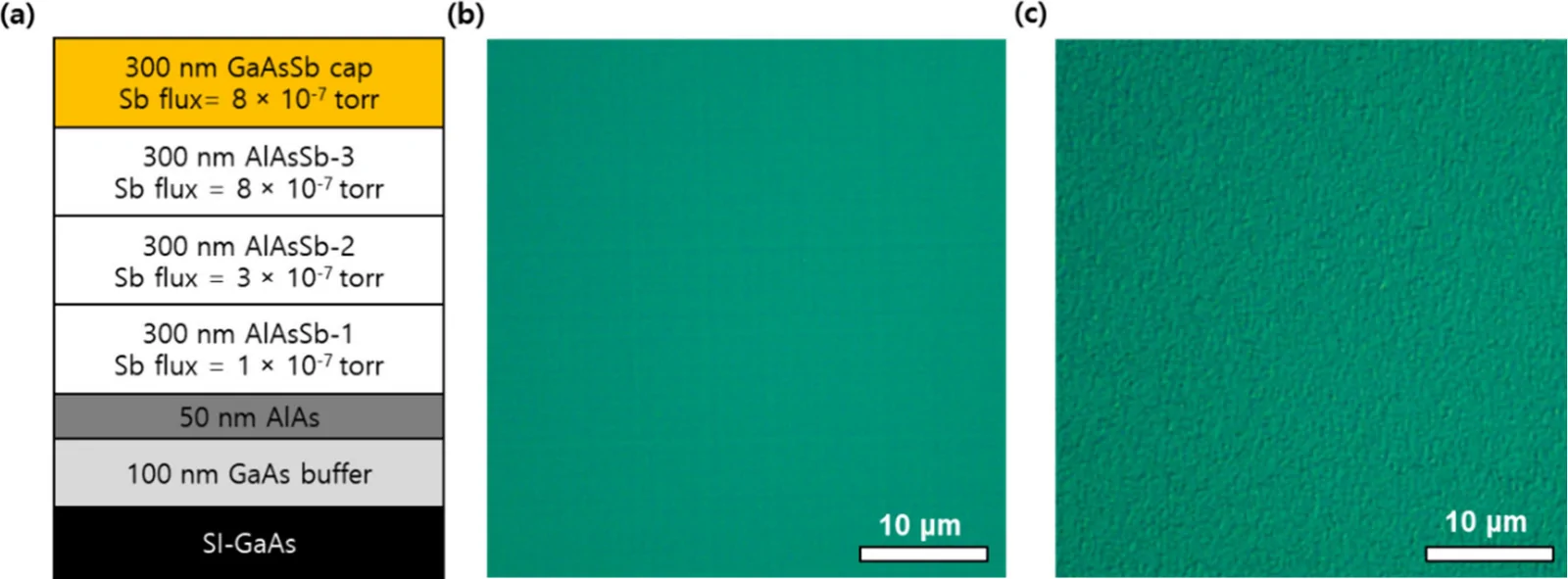
Sample structure and optical microscope images of high-temperature and low-temperature samples. (a) Structure of samples used in this study. Sb flux is measured using a beam-equivalent pressure gauge prior to MBE growth. OM images of samples grown at (b) 635 °C and (c) 535 °C.

The structure was then completed with a 300 nm GaAsSb layer to prevent AlAsSb from oxidation and to compare Sb incorporation into GaAsSb with different growth temperatures. All samples were grown at different growth temperatures, from 535 to 635 °C, with a 25 °C step between the samples, measured by a thermocouple to investigate the growth window boundaries of AlAsSb.

## 3 Results and Discussion

Optical microscope (OM) images of the films grown at 635 and 535 °C are shown in Figure 1(b) and 1(c), respectively. It can be clearly seen that the sample grown at high temperature shows a distinctly smooth surface with a clear cross-hatch pattern, while the sample grown at low temperature shows a very rough surface, indicating poor epitaxial quality or even not a proper epitaxial growth, suggesting that the film was simply deposited on the substrate. The OM images suggest that higher growth temperatures promote smoother surface morphology, however, assessment of epitaxial quality requires further structural characterization.

10 × 10 μm^2^ AFM images are shown in Figure 2 (a-d) for samples grown at 535 °C, 560 °C, 610, and 635 °C, respectively. A rough, amorphous-like surface can be observed for the sample grown at 535 °C, with a measured root-mean-square (RMS) surface roughness of 11.5 nm. Such a high RMS value is not typical for epitaxial growth. Based on this surface analysis, we conclude that 535 °C is too low for successful AlAsSb epitaxy on GaAs substrates. The poor quality of the films grown at 535 °C may be related to insufficient adatom mobility, sticking coefficients, and the complex incorporation kinetics of As and Sb during low-temperature growth. A detailed investigation of these mechanisms will be the subject of future study.

**Figure 2:**
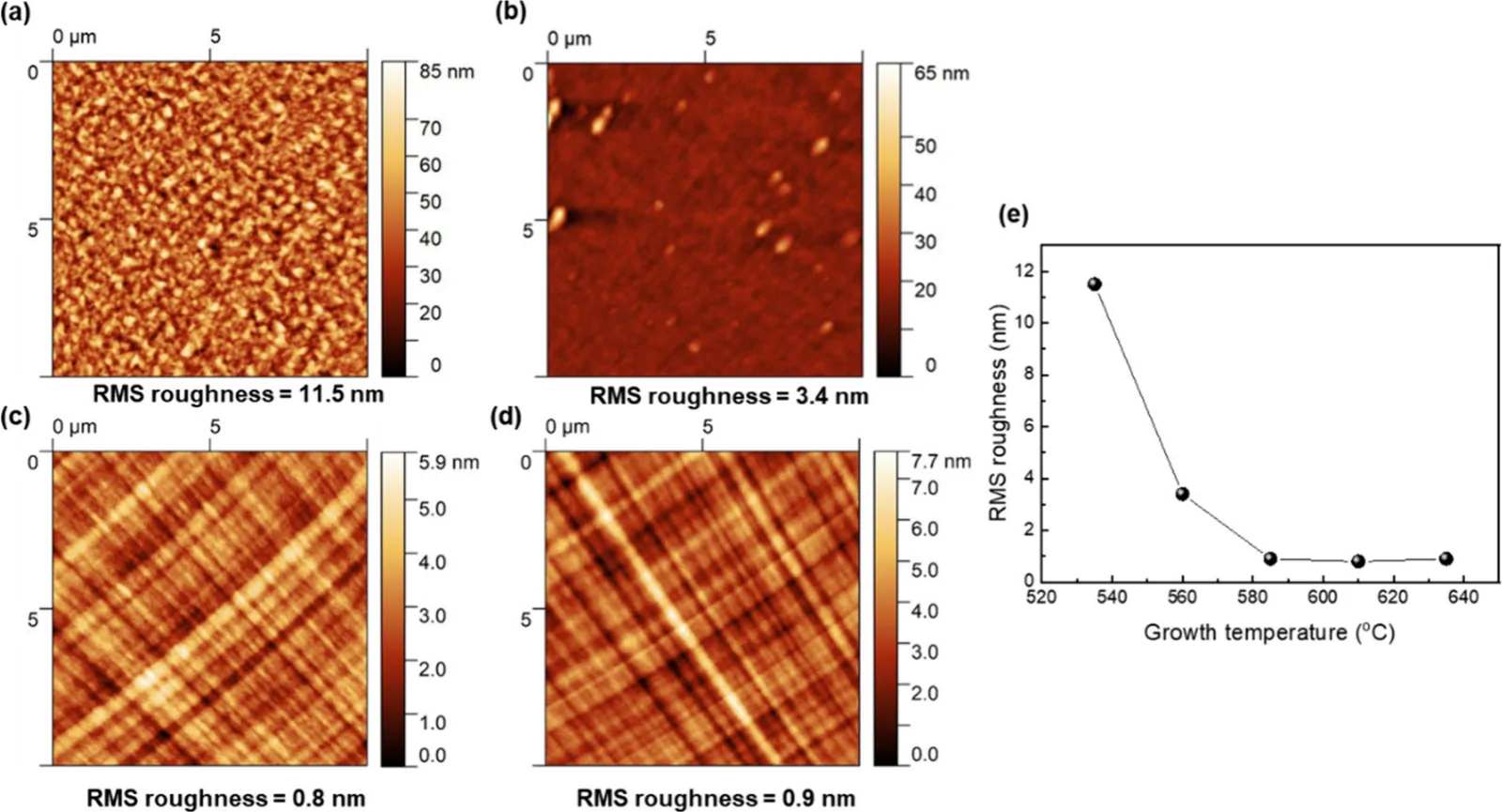
Atomic force microscope images of samples grown at (a) 535 °C, (b) 560 °C, (c) 610 °C, and (d) 635 °C. (e) Root-mean-square roughness dependence on growth temperature shows that higher growth temperature is beneficial for achieving smooth AlAsSb film growth.

The sample grown at 560 °C exhibited a relatively rough surface with an improved RMS roughness of 3.4 nm. From AFM scans, it is difficult to identify where the growth degradation occurs, since the AFM images correspond to the GaAsSb cap surface. However, GaAsSb is a well-known epitaxial material with a wide growth window, and low-temperature epitaxial GaAsSb has been reported.^24,25^ Therefore, the observed roughness could be attributed to the underlying AlAsSb layers, which suffer degradation when grown at a low temperature. By increasing the growth temperature to 585 °C and above, the surfaces become exceptionally smooth, with RMS roughness values as low as 0.9 and 0.8 nm for the samples grown at 585 and 610 °C, respectively. The AFM scans clearly show the emergence of a cross-hatch pattern, which is characteristic of misfit dislocation arrays formed during lattice-mismatched epitaxy.^26^ This is expected in our structure, since higher Sb incorporation increases the lattice mismatch. Thus, the formation of a cross-hatch pattern is consistent with successful Sb incorporation, whereas its absence would indicate homoepitaxial growth of GaAs without significant Sb incorporation. We summarized the AFM surface roughness measurements in Figure 2(e). A clear trend can be observed where, as the temperature increases, the surface morphology improves significantly. However, at 635 °C, the surface roughness slightly increases, suggesting that growth temperatures beyond this point may be too high for optimal AlAsSb growth.

Figure 3 shows the symmetric (004) and asymmetric (115) RSMs of our samples. The RSMs were measured to extract the strain relaxation, film tilt, and composition parameters of the GaAsSb/AlAsSb layers. The reciprocal lattice units were defined according to the equations reported elsewhere.^27^ Figure 3(a-b) shows the (004) and (115) scans of the sample grown at 535 °C. Only a single peak is observed, supporting our hypothesis that most of the material was not epitaxially grown and that the deposited layer is largely amorphous or polycrystalline on the GaAs substrate.^28^ Figure 3(c-d) shows the scans for a sample grown at 560 °C. A distinctive bottom-most peak corresponds to the GaAsSb cap, confirming that the Sb species incorporation rate increased by changing the group-III element from Al to Ga. However, the AlAsSb peaks are merged, and the three individual layers cannot be precisely distinguished. We assume that the layer with the highest Sb composition was not grown properly, which explains the absence of a corresponding RSM peak. This interpretation is consistent with the AFM results, where the surface roughness improved but remained relatively high. Figure 3(e-f) shows the RSM scans of the sample grown at 585 °C. In this case, three distinct AlAsSb peaks corresponding to different Sb compositions are clearly observed, in addition to the bottom-most GaAsSb peak. A clear film tilt of −0.16, −0.25, and −0.35 degrees for the AlAsSb layers and −0.76 degrees for the GaAsSb layer is visible, and the well-defined peak positions indicate the high epitaxial quality of the structure. Additional verification of the epitaxial layer structure and interface quality can be performed using high-resolution X-ray diffraction and transmission electron microscopy, as demonstrated in previous studies.^29–31^ Figure 3(g-h) displays the scans of the sample grown at 610 °C. Here, the AlAsSb peaks begin to merge again, and the GaAsSb peak is closer to the AlAsSb peak compared to that of the 585 °C sample. This suggests that at higher temperatures, the increased Sb incorporation rate in GaAsSb is reduced. At 635 °C, Figure 3­(i-j), the RSMs reveal a highly mosaic structure. The GaAsSb peak shows signs of strong phase separation, and distinguishing the peaks of individual layers becomes difficult. Based on these observations, we conclude that the optimal growth temperature for AlAsSb layers in our structure is around 585 °C. Rocking curve scans can provide additional insights into the material grown for this study by measuring the full width at half maximum values of each peak. Currently, we do not have the samples studied for further analysis, but this could be the subject of future work by regrowing the materials.

**Figure 3:**
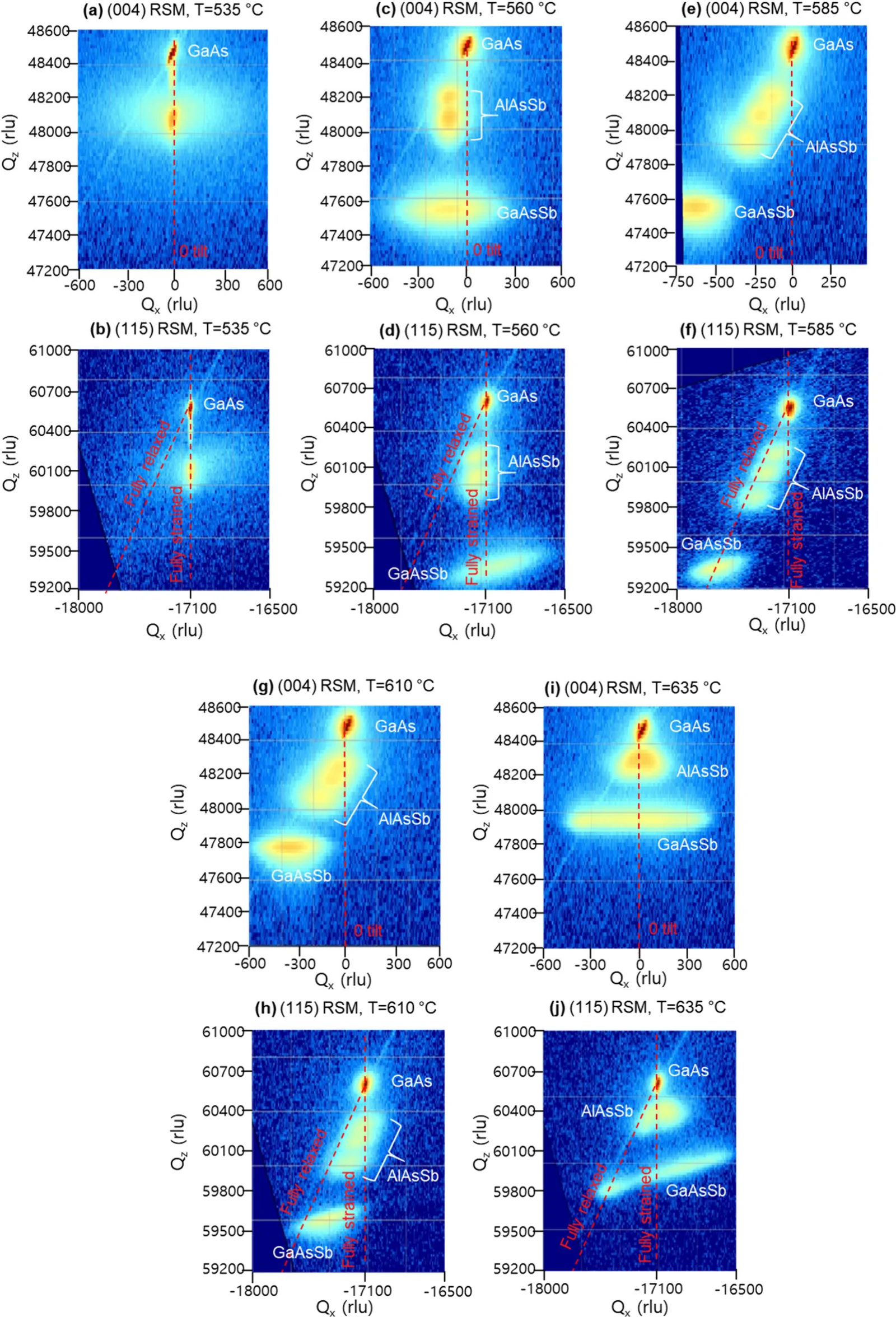
Symmetric (004) and asymmetric (115) reciprocal map scans for films grown at 535 °C (a-b), 560 °C (c-d), 585 °C (e-f), 610 °C (g-h) and 635 °C (i-j).

Figure 4 shows the film tilts, relaxations, and Sb compositions extracted from the RSM scans. The equations used for calculations can also be found elsewhere.^6,27^ Figure 4(a) shows the dependence of the film tilt over the growth temperatures, measured for the GaAsSb and the most Sb-rich AlAsSb layers. The most significant film tilt was observed at 585 °C for both layers. The GaAsSb layer experiences a significant tilt of up to −0.8 degrees, while AlAsSb is less tilted. A higher tilt can suggest lower relaxation of the films, since tilt can be linked to the presence of misfit dislocations in the film.^32^ While for the sample grown at 560 °C, the scan can be affected by a poor quality of the epitaxial growth, for structures grown at 585 and 610 °C, tilt can be extracted properly. Lower tilt for samples grown at 610 °C can suggest that a higher-temperature growth can improve the overall relaxation of the layers. This is shown in Figure 4(b), where the AlAsSb film has the highest relaxation degree at 610 °C. While the underlying AlAsSb layer gets more relaxed, its lattice constant increases, suggesting that the upper GaAsSb layer experiences more strain. This explains why the GaAsSb layer is less relaxed at 610 °C. Figure 4(c) shows the incorporation efficiency of Sb into AlAsSb layers with three different Sb fluxes used during the growth. It can be clearly seen that Sb incorporation exhibits a strong dependence on growth temperature. A simple flux-ratio model would predict Sb compositions of approximately 3.2%, 9.1%, and 21.1% for the investigated Sb fluxes. The measured compositions deviate significantly from these values, highlighting the nonlinear nature of Sb incorporation in the GaAsSb/AlAsSb system. The highest incorporation efficiency is obtained at a growth temperature of 585 °C. As growth temperature increases beyond 585 °C, the overall Sb incorporation decreases. This phenomenon also explains why the largest tilt and lower relaxation can be observed for a film grown at 585 °C compared to 610 °C. The observed decrease in Sb incorporation with increasing growth temperature is consistent with previous reports on Sb-containing III–V films.^25^ This behavior has been attributed to a reduction in the effective Sb sticking coefficient, enhanced Sb desorption, and As–Sb exchange reactions at the growth surface, all of which reduce the amount of Sb incorporated into the growing film.

**Figure 4:**
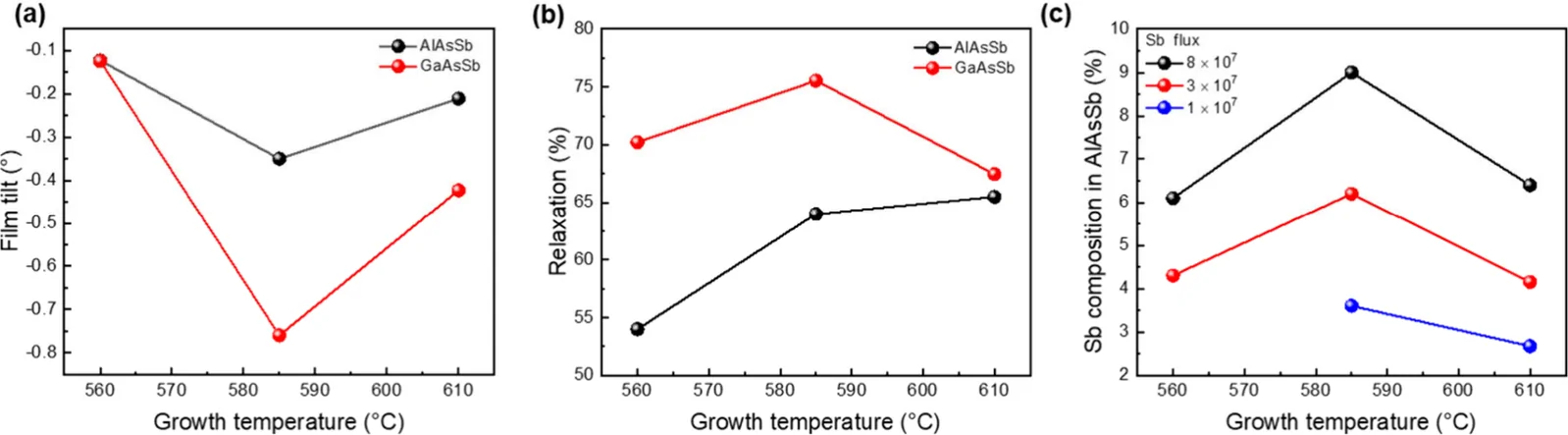
X-ray diffraction analysis of the grown structures. (a) Film tilt of GaAsSb and AlAsSb extracted from (004) RSM scan dependence on growth temperature. (b) Relaxation degree of AlAsSb and GaAsSb films extracted from (115) RSM scan. In (a) and (b), the AlAsSb layer has the highest Sb concentration. (c) Sb composition variation dependence on growth temperature of three AlAsSb layers.

Photoluminescence (PL) spectra are presented in Figure 5(a) to evaluate the composition of the GaAsSb cap layer and to compare the material quality of the samples grown at different temperatures. PL measurements were performed at room temperature using a 532 nm green laser as the excitation source. For the samples grown at 535 and 560 °C, almost no PL emission was detected, consistent with the earlier AFM and RSM results and confirming the poor quality of films grown at relatively low temperatures. As the growth temperature increases, the PL intensity increases, and the emission peak width decreases, indicating an improvement in material quality. The observed intensity variations can be linked to the composition of the underlying AlAsSb layers. In particular, the sample grown at 585 °C exhibited the highest Sb incorporation in AlAsSb. As a result, the overlying GaAsSb layer experienced increased strain, leading to reduced PL intensity despite otherwise good structural quality. The strongest PL intensity was observed for the sample grown at 610 °C, where Sb incorporation into the AlAsSb layer decreased, lowering the overall strain in the GaAsSb cap. As the temperature increases, Sb composition in the underlying AlAsSb layers decreases, resulting in improved optical properties of the capping GaAsSb layer. The sample grown at 635 °C exhibited the narrowest emission peak among all samples, which is counterintuitive to the RSM analysis that revealed poor Sb incorporation into the underlying AlAsSb layers and strong phase separation. The strong PL emission observed for the sample grown at 635 °C may be related to carrier localization within compositionally nonuniform regions formed during phase separation. Similar localization effects induced by compositional fluctuations have been reported in InGaN/GaN quantum-well structures.^28,33^ The Sb compositions extracted from PL and from RSM analysis are summarized in Figure 5(b). The Sb composition of the GaAsSb layer was estimated from the measured PL peak energy using the reported bandgap-composition relationship for GaAsSb alloys.^8^ The small differences between the PL-derived and RSM-derived compositions are expected, since the two techniques measure composition through different material properties. While RSM determines composition from lattice parameters, PL estimates composition from the bandgap, which can additionally be affected by residual strain, defects, and surface morphology. The Sb incorporation into GaAsSb decreases with increasing growth temperature, with the maximum Sb composition obtained for the sample grown at 560 °C. However, since the 560 °C sample exhibited poor AlAsSb layer quality and rough surface morphology, we conclude that the optimal growth temperature for the GaAsSb/AlAsSb system is 585 °C. Although the highest PL intensity is observed at 610 °C, the optimal growth temperature of AlAsSb was defined based on the maximum Sb incorporation achieved in the AlAsSb layers, which occurs at 585 °C. The optimal growth temperature corresponds to the temperature yielding the highest Sb incorporation, whereas the growth window refers to the broader temperature range (580–620 °C) over which high-quality epitaxial AlAsSb layers can be obtained. Within the AlAsSb growth window (580–620 °C), a consistent trend was observed: when switching from GaAsSb to AlAsSb under the same Sb flux, the Sb incorporation efficiency decreases by approximately a factor of 2. These results clarify the temperature- and flux-dependent behavior of Sb incorporation in the GaAsSb/AlAsSb system.

**Figure 5:**
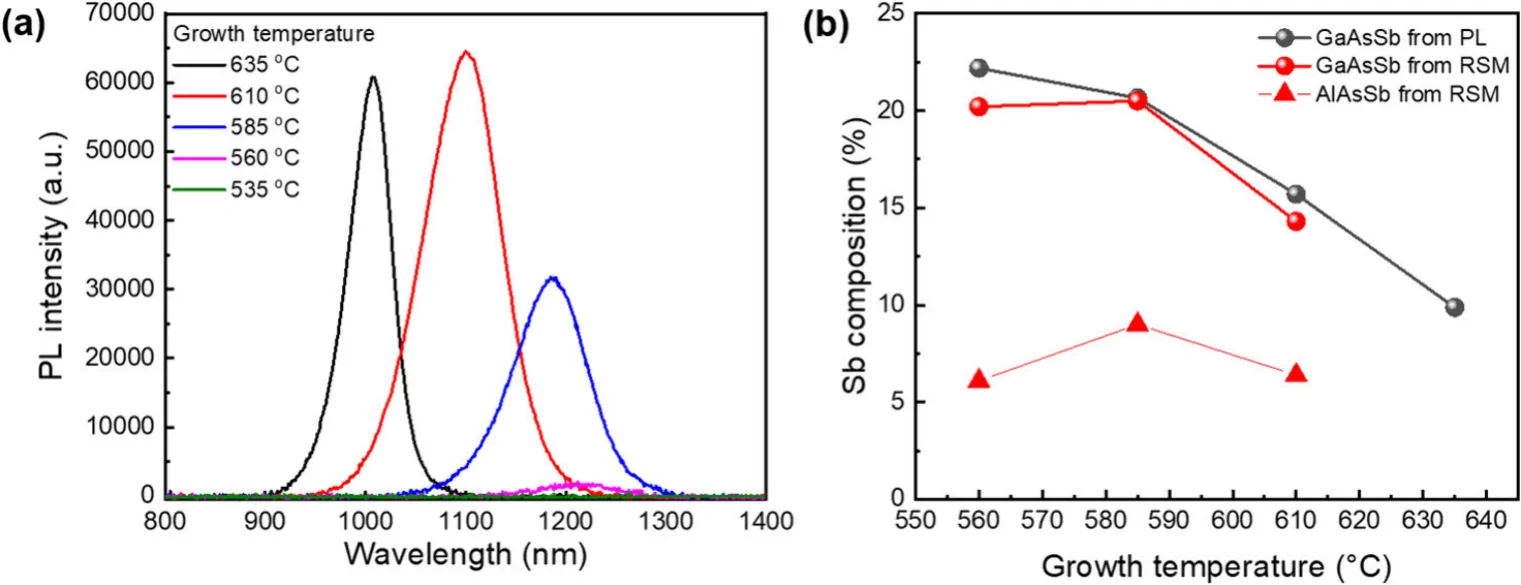
(a) Photoluminescence (PL) of the as-grown structures. (b) Compositional data of GaAsSb extracted from the PL measurement plotted together with RSM. AlAsSb data extracted from RSM are also plotted for comparison.

## 4 Conclusions

In this work, we investigated the growth of high-quality AlAsSb/GaAsSb layers on GaAs substrates via molecular beam epitaxy. We determined that the optimal growth temperature, and thus the growth window for AlAsSb layers, lies between 580 and 620 °C. Within this range, smooth surfaces and well-defined RSM peaks were obtained, with a maximum Sb incorporation efficiency at 585 °C. At lower temperatures, AlAsSb growth suffers from severe surface degradation, and no epitaxial growth was achieved. At higher temperatures, although the overall surface morphology remains smooth, Sb incorporation decreases to a minimum and degradation of the layers occurs, including strong GaAsSb phase separation. The Sb composition increased from 9% in the AlAsSb layer to 20.5% in the GaAsSb layer at 585 °C, suggesting a dramatic change in the As and Sb incorporation dynamics. These results provide valuable insights for optimizing the MBE growth of AlAsSb and GaAsSb for SWIR and MWIR optoelectronic applications.
